# Two-way association between alopecia areata and sleep disorders: A systematic review of observational studies

**DOI:** 10.1016/j.amsu.2022.104820

**Published:** 2022-11-05

**Authors:** Syeda Tayyaba Rehan, Zayeema Khan, Hussain Mansoor, Syed Hasan Shuja, Mohammad Mehedi Hasan

**Affiliations:** a Dow University of Health Sciences, Karachi, Pakistan; b Department of Biochemistry and Molecular Biology, Faculty of Life Science, Mawlana Bhashani Science and Technology University, Tangail, 1902, Bangladesh

**Keywords:** Alopecia areata, Sleep disorder, Insomnia, Baldness, Sleep apnea

## Abstract

**Background:**

Alopecia Areata (AA) is found to be the most prevalent autoimmune disorder amongst the general population. It was observed that AA patients are at a significantly higher risk of developing obstructive sleep apnea and non-apneic insomnia than patients without AA. On the contrary, patients with identified sleep disorders were found to be more prone to developing AA as compared to the patients without sleep disorders. This study, therefore, validated the hypothesis of a bidirectional association between AA and sleep disorders.

**Aims:**

In this systematic review, our primary aim is to assess the prevalence of sleep disorders in Alopecia Areata patients while also assessing the inverse relationship between the two disorders.

**Methods:**

A literature search of MEDLINE, Google Scholar and Cochrane CENTRAL was performed from their inception to April 2022. Articles were selected for inclusion if they met the following eligibility criteria: (a) Studies enrolling patients having alopecia areata to assess the sleep quality. (b) Studies assessing the risks of alopecia areata in individuals with sleep disorder (c) Studies evaluating the bidirectional association between alopecia areata and sleep quality. Case reports, commentaries, and editorials were excluded. The outcomes of recruited studies were qualitatively synthesised and study findings are summarized in the results section and tabulated in summary tables.

**Results:**

Our search on electronic databases yielded 1562 articles. After abstract screening and full text review, 5 cross sectional and 3 cohort studies are included in this systematic review. Cases with PSQI scores higher than 5 and 6 were found to be in greater numbers amongst the AA patient population when compared to the control population (*p* < 0.001). Moreover, studies showed that patients with sleep disorders were greatly predisposed to develop subsequent AA as compared to patients without sleep disorders (aHR 4.70; 95% CI 3.99–5.54) (P < 0.0001).

**Conclusion:**

The findings from our results display a significant bi-directional cause-effect relation between AA and sleep disorders. However, more large-scale observational studies on this subject are required to further validate our findings.

## Highlights

• Alopecia Areata (AA) is found to be the most prevalent autoimmune disorder amongst the general population.
• It was observed that AA patients are at a significantly higher risk of developing obstructive sleep apnea and non-apneic insomnia than patients without AA.
• In this systematic review, our primary aim is to assess the prevalence of sleep disorders in Alopecia Areata patients while also assessing the inverse relationship between the two disorders.
• The findings from our results display a significant bi-directional cause-effect relation between AA and sleep disorders.

## 1 Introduction

Alopecia Areata (AA) is a non-inflammatory, autoimmune disease that presents as bald patches on hair-bearing areas of the body, especially the scalp [1]. It can be subdivided into alopecia totalis and alopecia universalis, which refer to hair loss from the scalp and body, respectively [2,3]. According to a systematic review from 2015, the lifetime incidence of AA was reported to be around 2% worldwide. The overall prevalence of AA was found to be the highest amongst all autoimmune disorders and the second highest amongst all hair loss disorders, only falling below androgenic alopecia. Furthermore, contrary to popular belief, data from the two population studies included in the systematic review revealed that the difference in the incidence of AA amongst male and female populations was not significant [4].

Qualitatively, AA has been found to be associated with several comorbidities including skin conditions such as vitiligo and atopic dermatitis. Disorders such as inflammatory bowel disease, psoriasis, systemic lupus erythematosus (SLE), and rheumatoid arthritis have also been found to be closely linked to AA [[5], [6], [7], [8], [9]]. Moreover, numerous studies have attempted to analyze the incidence of psychiatric illnesses in AA patients. The results of such studies support the link between AA and major psychiatric illnesses such as depression, anxiety, social phobias, paranoia, alexithymia and sleep disturbances [[10], [11], [12], [13], [14]]. The estimated incidence of psychiatric illnesses in individuals with AA is somewhere around 66%–74% [4].

In recent years, evidence regarding a link between AA and sleep disorders has been piling up. It has been hypothesized that this association is bi-directional. A 2020 population-based cohort study from Taiwan investigated this relationship between AA and sleep disorders in a large sample population. It was reported that the risk of developing obstructive sleep apnea (OSA) and non-apnea insomnia was significantly higher in AA patients as compared to patients that did not have AA. On the contrary, patients with identified sleep disorders were found to be more prone to developing AA as compared to patients without sleep disorders. This study, therefore, validated the hypothesis that a bidirectional relationship exists between AA and sleep disorders [15]. In this systematic review, our primary aim is to analyze the incidence of sleep disorders in AA patients while also assessing the inverse relationship between the two disorders.

## 2 Methods

This systematic review was performed in line with the Preferred Items for Systematic Review and Meta-Analysis (PRISMA) guidelines [16]. To evaluate the methodology of the study AMSTAR checklist has been included in the supplementary file [17].

### 2.1 Data sources and search strategy

We conducted a comprehensive literature search of MEDLINE, Google Scholar, and Cochrane CENTRAL from their inception to April 2022 by using medical subject headings (MeSH) ‘alopecia areata’ OR ‘alopecia’ OR ‘baldness’ AND ‘sleep disorders’ OR ‘sleep disturbances’ OR ‘sleeplessness’ OR ‘insomnia’ OR ‘sleep apnea’ with no time, language and sample size restrictions. The search string has been subsequently altered and reoriented in each search engine. The complete search strategy used in each of the databases is given in Supplementary Table S1. Furthermore, to identify grey literature, reference lists of relevant studies, and online libraries such as clinicaltrials.gov and preprint servers like medrvix were also screened.

### 2.2 Study selection

The articles retrieved from the systematic search were exported to EndNote Reference Manager (Version X7.5; Clarivate Analytics, Philadelphia, Pennsylvania), where duplicates were screened for and removed. The remaining articles were carefully assessed by two independent reviewers (SHS and STR) at the title and abstract level, after which the full text was comprehensively reviewed to affirm relevance. Studies were selected if they met the following pre-defined inclusion criteria: (a) Studies enrolling patients having alopecia areata to assess the sleep quality. (b) Studies assessing the risks of alopecia areata in individuals with sleep disorder (c) Studies evaluating the two-way association between alopecia areata and sleep quality.

Duplicate records and articles in languages other than English were excluded. Case reports, commentaries, and editorials were also excluded.

### 2.3 Data extraction

The following information was extracted on a standard excel sheet: study type, study year, sample size, age, and gender from the eligible articles. The primary outcome of interest was the sleep disturbances owing to a diagnosis of alopecia areata while the secondary outcome was the risk of occurrence of new-onset alopecia areata due to a predisposing sleeping disorder.

### 2.4 Data synthesis

The outcomes of recruited studies were qualitatively synthesised and not combined for meta-analysis due to the different clinical and methodological approaches used in the studies. Study findings are summarized in the results section and tabulated in summary tables.

### 2.5 Quality assessment

For cohort, cross-sectional, and case-control studies, two investigators (Z.K and S.T.R) independently assessed the quality of the eight included studies using the Newcastle-Ottawa scale (NOS). Disagreements on risk of bias assessments between the two review writers (Z.K and S.T.R) were resolved via discussion with a third review author (H.M). Quality assessment was performed using NOS for cohort and case-control studies [18]and for cross-sectional studies, an adapted version of NOS was acquired from Herzog et al.‘s study [19] This scale assigns grades to studies based on three factors (selection, comparability of study groups, and the outcome of interest). A study can receive a highest rating of 9 for cohort studies and a maximum possible score of 10 for cross-sectional studies, with 9 representing the best study. Cross-sectional studies scoring 9 or 10 points were thought to have a low risk of bias; 8 points were thought to have a medium risk of bias; and 6 points or fewer were thought to have a high risk of bias. Similarly, studies with a total score of 8 or 9 points were deemed to have a low risk of bias; studies with a score of 7 or 6 points were judged to have a moderate risk of bias; studies with a score of 5 points or less were regarded to have a high risk of bias.

## 3 Results

### 3.1 Literature search

**Fig. 1:**
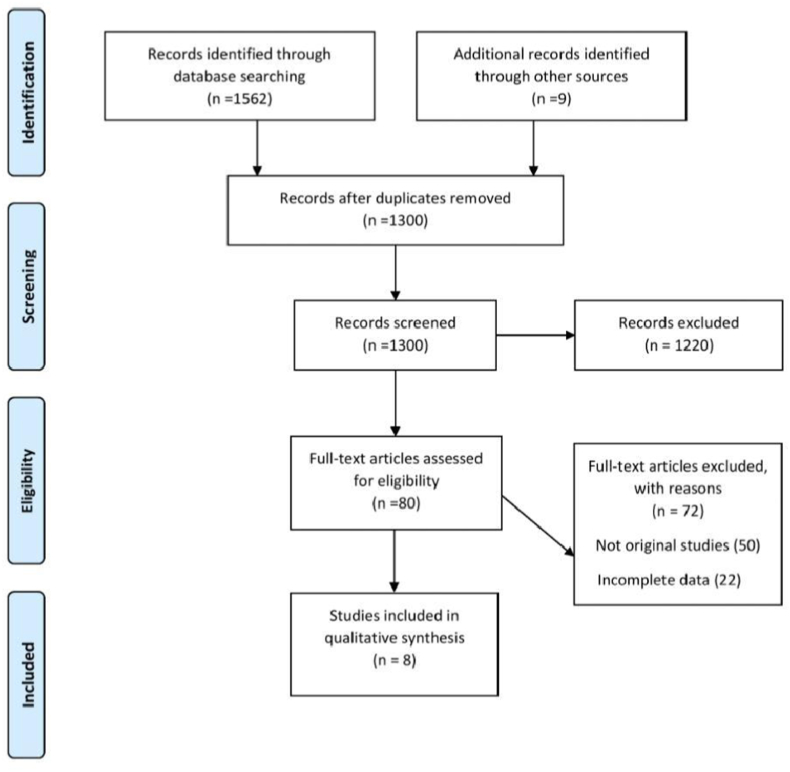
Summary flow chart of literature search.

Our search on electronic databases resulted in 1562 articles. After removing duplicate articles and abstract screening, 80 articles were selected for full text review. 72 studies were further removed after full text review that did not meet the inclusion criteria. Finally, 5 cross-sectional and 3 cohort studies are included in this systematic review. Summary of the literature search is presented in Fig. 1.

### 3.2 Quality assessment

Four cross-sectional studies, three cohort studies, and one case-control research were evaluated for bias; the 8 cross-sectional, cohort, and case-control studies all showed a medium risk of bias. One major risk of bias in all cross-sectional studies which failed to report the number of non-respondents [20,21,23,24]. Shakoei et al. did not employ a sizable patient population [20]. The failure to report the number of individuals lost due to follow-up was one main bias observed in all cohort studies [1,15,25]. Similarly, absence of non-response rate in case-control studies was the only bias in the study conducted by Otzekin et al. [23]. Summary of quality assessment is presented in **supplementary file 2**.

### 3.3 Study characteristics

Six of the eight studies are looking at our primary outcome, which is sleep disruptions caused by alopecia areata [1,[20], [21], [22], [23], [24]]. One study is looking into the likelihood of developing new-onset alopecia areata as a result of a predisposing sleeping problem [25]. Dai et al. is evaluating both primary and secondary outcomes [15]. The Epworth Sleepiness Scale (ESS), which evaluates Excessive Daytime Sleepiness (EDS) using a self-administered questionnaire developed to evaluate EDS using a score system, was used in two investigations to assess sleep quality in the patient group [20,21]. Scores equal to or more than 11 are considered to be EDS, any score lower is considered as non-EDS [21]. Similarly, three studies used the Pittsburgh Sleep Quality Index (PSQI), a self-reported questionnaire that assesses sleep latency, sleep length, habitual sleep efficacy, sleep disruptions, sleeping medicine use, and daytime dysfunction using a global PSQI score of 0–21 to evaluate sleep quality [20,22,23]. Dai et al. examined sleep disorders utilising ICD-9-CM codes, such as Obstructive Sleep Apnea (OSA) (327.2, 780.51, 780.53, 780.57) and non-apnea insomnia (327.2, 780.51, 780.53, 780.57). (307.41, 307.42, 780.52) [15]. Sorour et al. used the Diagnostic and Statistical Manual of Mental Disorders, 5th edition to assess sleep disorders (DSM-5) [24].

Kim et al. did not use a scale to measure sleep problems; instead, individuals with two main International Classification of Diseases, Tenth Revision diagnosis codes issued by board-certified psychiatrists were classified as having psychiatric illnesses [1]. Various assessment procedures were utilized in the two studies that evaluated the secondary outcome [15,25]. Seo et al. used ICD-10 codes to evaluate alopecia areata diagnosis (L63) [25]. For the assessment of AA, Dai et al. did not mention any assessment method [15].

Sleep quality was assessed in four trials [[20], [21], [22], [23]]. Shakoei et al. looked at sleep disturbances as well [20]. Two studies looked at the link between AA and sleep by tracking the prevalence of sleep disorders in AA patients [15,24]. Kim et al., looked at nonorganic sleeping problems in particular [1].

### 3.4 Primary outcome

Sleep disruption was assessed using ESS and PSQI scores in a cross-sectional study conducted by Shakoei et al. [20]. The PSQI score was considerably higher in the patient group (p < 0.001), and a question assessing patients' assessments of their sleep quality revealed that the AA case group had a lower estimate of their sleep quality than controls (p < 0.001) [20]. When comparing the AA patient population to the control population, cases with PSQI values higher than 5 and 6 were detected in increased numbers (p < 0.001) [20]. Further analysis revealed a strong relationship between PSQI scores and patient self-reported sleep quality (Spearman's = −0.762, p < 0.001) [20].

Shakoei et al. was the only study that looked into a possible link between anxiety and sadness in AA patients and sleep quality is [19]. The PSQI score among AA patients with anxiety and depression was considerably higher (9.9 ± 5.28 vs. 4.76 ± 3.08, p = 0.001) [20]. When self-assessment of sleep quality was further assessed, it was shown that it was lower in AA patients with anxiety (2.10 ± 0.99 vs. 3.32 ± 1.08, p = 0.002) [20]. Oztekin et al. [22] evaluated sleep quality using a PSQI scale, and the results were identical to those of Shakoei et al.‘s study [20]. Sleep latency, sleep disturbance, Subjective sleep quality, and daytime dysfunction subscale scores were all statistically higher in the study group as compared to the control group (p = 0.002, p = 0.001, p < 0.001, and p < 0.001, respectively) [22]. Alopecia areata affected 14 persons out of the total patient population of 70 [23]. This study looked at seven different PSQI questionnaire assessments [23]. Sleep latency, sleep duration, sleep efficiency, sleep interruptions, sleeping medicine usage, and daytime dysfunction were all considered (Mean ± SD; 1.31 ± 0.85, 1.43 ± 1.09, 0.92 ± 0.95, 0.18 ± 0.40, 1.07 ± 0.27, 0.50 ± 0.94, 1.21 ± 1.05 correspondingly) [21]. The importance of these findings have not been reported in this study [23].

The ESS scale was also used by Shakoei et al. to assess Excessive Daytime Sleepiness (EDS). Although there was no statistically significant difference in mean ESS scores between the case and control groups (p = 0.34), the number of cases with an ESS score of 11 was found to be higher in the patient group 15 (71.4%) than in the control group 6 (28.6%), (p = 0.02) [20]. Inui et al. also assessed EDS using the proposed ESS scale [21]. The findings of the ESS revealed that 12 of the 105 individuals in the case group (11.4%) had substantial EDS due to a score greater than 10 [21]. The mean EDS score was 5.657 ± 3.932 (mean ± SD), indicating that AA patients have generally good sleep quality [21]. ICD-9 codes were used to assess sleep quality and disruption in Dai et al.‘s study [15]. The findings revealed that 483 AA patients (45 cases of obstructive sleep apnea (OSA) and 438 cases of non-apneic insomnia) acquired sleep problems [15]. When compared to control persons, AA patients showed a higher incidence of developing OSA and non-apneic insomnia (aHR 3.80; 95% CI 2.53–5.71) and (aHR 4.20; 95% CI 3.68–4.79), respectively [15].

To assess sleep disorders and disruption, Sorour et al. conducted a psychiatric evaluation using the Diagnostic and Statistical Manual of Mental Disorders, Fifth Edition (DSM-5). When the 208 AA patients were assessed, 5 of them (2.40%) experienced sleep disturbances [24]. In Kim et al.‘s study, following the evaluation, 42 of the 7706 AA patients acquired nonorganic sleep problems (aHR 1.59; 95% CI 1.11–2.27), which was a statistically significant finding (p = 0.011) (Table 1) [1].

**Tabel 1:** Study characteristics table (Primary outcome).

Author (Year)	Study Design	Number of Participants (N, age,sex)	Participant Selection	Control	Place of Study	Assessment Tool	Findings
Shakoei 2022 [20]	Cross-sectional study	102 (51 = control,51 = case group),Male = 24, Female = 27 29.71 ± 10.78 years	Dermascopy confirmed Alopecia Areata patients	Healthy individuals with no Alopecia Areata diagnosis	Tehran University of Medical Sciences	PSQI and ESS scales	Mean PSQI score and ESS score, significantly higher in AA population (*p* < 0.001, P = 0.02; respectively)
Ying-Xiu Dai 2020 [15]	Bidirectional cohort study	5648; Male = 2,946, Female = 2,70234.1 (26.8–43.5) years	Diagnosed AA patients ≥20 years, identified by ICD-9-CM code 704.01 confirmed three times by board-certified dermatologists	Age, gender, monthly premium, residency status, and comorbidities were all matched in the control group.	Taiwan	ICD-9-CM codes	A significant number of patients with AA developed OSA and non-apnea insomnia (aHR 3.77; 95% CI 2.48–5.73), (aHR 184 4.01; 95% CI 3.49–4.59), respectively
Oztekin 2020 [22]	Cohort study	52 = Case group, 51 = control group; 28.8 ± 7.7 years	Patients above the age of 18 who were admitted to a dermatology clinic and diagnosed with alopecia areata.	Healthy volunteers who volunteered to participate in the study and had no history of mental, systemic, or dermatological disorders.	Hitit University	PSQI Scale	Significant difference was found between AA case group and control group, with AA patients experiencing more sleeping disorders
Kashaninasab 2020 [23]	Cross-sectional study	70; 30 = Men, 40 = Women; 32.74 ± 9.97 years	Types of alopecia were clinically identified and classified by dermatologists	N/A	Dermatology Clinic of Rasoul-e-Akram Hospital	PSQI scale	The PSQI scale assessments were measured. Significance of the findings are not reported
Kim 2019 [1]	Retrospective cohort study	7706 = case group; 30,824 = control; age not specified	Only dermatologists assigned two main International Classification of Diseases, Tenth Revision, diagnostic codes for AA to patients with AA.	Patients who had never been given an AA diagnosis and were matched 1:4 (n = 30,824) by index date, age group, sex, and income.	Korea	Principal International Classification of Diseases, Tenth Revision	A significant number of AA patients developed nonorganic sleeping disorders (p = 0.011)
Sorour 2016 [24]	Cross-sectional study	208; Male = 122, Female = 186; 17–60 years	Male and female patients aged 17–60 years old with a persistent dermatologic illness for more than 6 months were recruited.	N/A	Al-Haud Al-Marsoud Hospital, Al-Husain Al-Azhar University Hospital, Bab Al-Shariah Al-Azhar University Hospita**l**	PSQI scale	2.40% AA patients developed sleep disorders
Inui 2014 [21]	Questionnaire- based study	105; Male = 33, Female = 72; 16–74 years,	After obtaining written consent forms, one hundred and five patients with AA were enrolled at Shin-Osaka Clinic.	N/A	Japan	ESS scale	11.4% of the patient group with AA developed experienced sleeping disturbances

### 3.5 Secondary outcome

Seo et al. looked at the risk of getting AA as a result of a sleep disturbance. When comparing the AA population to the control population, the findings revealed a considerable risk (crude HR 1.610 [95% CI 1.350–1.919]) [25]. HR sensitivity was unaffected by sensitivity analyses based on age, gender, geography, income, and comorbidities (adjusted HR 1.651 [95% CI 1.382–1.974]) [25]. In patients with sleeping difficulties, the risk of acquiring AA is considerably higher than in the general population (log-rank p < 0.001) [25]. Seo et al. also did subgroup analysis to further assess the final result [25]. The age group (0–24 years old) had a substantially greater incidence of alopecia areata (2.67 per 1000 person-years, adjusted HR 2.605 [95% CI 1.699–3.992]) [25]. The second age group (25–44 years old) had a comparable significant finding (adjusted HR 1.765]95% CI (1.357–2.295)] (log-rank p < 0.001) [25]. On the other hand, on the basis of AA risk (P = 0.181) (P = 0.252), the older age groups (44–65 years and 65 years) did not differ substantially from the control groups [25].

Alopecia areata affected 308 people out of 93,130, according to Dai et al., including 7310 patients with OSA and 85,820 patients with non-apnea insomnia) [15]. This sample included 20 individuals with OSA and 288 patients with non-apneic insomnia [15]. Patients with sleep disorders were shown to be considerably more likely than those without sleep disorders to develop subsequent AA (aHR 4.70; 95% CI 3.99–5.54) (P < 0.0001) [15]. OSA and non-apnea insomnia are both connected to a higher risk of developing AA (aHR 3.89; 95% CI 2.46–6.16) and AA (aHR 4.77; 95% CI 4.03–5.64), according to subgroup analyses [15] (Table 2).

**Table 2:** Study characteristics table (secondary outcome).

Author (Year)	Study Design	Number of Participants (N, age,sex)	Participant Selection	Control	Place of Study	Duration	Findings
Dai et al. [15]	Bidirectional cohort study	Case = 93,130; Control = 372,520; Male = 46,57, Female = 46,554; 43.6 years	Patients over the age of 20 who suffer from sleep disorders	After matching for age, sex, monthly premium, residence status, and comorbidities, four persons were chosen as research controls for each patient with sleep disturbance.	Taiwan	Not specified	Patients with OSA and non-apneic insomnia significantly developed AA (P < 0.0001)
Seo et al. [25]	Retrospective Cohort	Case = 25,800; Control = 129,000; Male = 10,544; Female = 15,256	Patients with F51 (sleep disorders not caused by a chemical or recognised physiological condition) or G47 (sleep disorders) ICD-10 codes were identified.	Control subjects matched for age and sex were selected randomly at a frequency of 1:5.	Korea	ICD-10 codes	Incidence ratio of sleeping disorders patient group developing AA was found to be significant log- rank *p* < 0.001

### 3.6 Relation of sleep disturbance and quality with the severity of alopecia areata

Inui et al. looked for a link between the severity of AA and sleep quality. The SALT score was used in this investigation to determine the severity of AA: 0, no hair loss; S1, 1–25% hair loss; S2, 26–50% hair loss; S3, 51–75% hair loss; S4, 76–99% hair loss; S5, totalis and universalis type AA [21]. The average SALT score was 2.59 ± 2.68, indicating that 64.8% of the hair on the scalp had been lost, suggesting the severity of the condition [21]. The ESS scores were not found to be substantially associated with the severity of AA after a Spearman rank correlation analysis [rs = −0.06]. (95% CI -0.24 - 0.138) [21]. Oztekin et al. used the SALT score to assess AA severity, but found no significant link between AA severity and sleep quality (p = 0.092) [22]. Further analysis of the PSQI score in AA patients with a poor prognosis revealed a substantial increase in the PSQI score in patients with AA for more than 5 years (p = 0.001) [22]. Shakoei et al. examined the severity of alopecia areata, but found no significant link between the development of sleeping difficulties and severe alopecia areata, ESS (r = 0.06, p = 0.64) or PSQI (r = 0.02, p = 0.85) scores [20].

### 3.7 Sex differences in relation with sleep quality and alopecia areata

Shakoei et al. discovered a substantially higher PSQI score in women when analysing sleep disruption (p = 0.02) [20]. When compared to the AA male patient population, ESS assessments in AA women were considerably lower (p = 0.04) [20]. Oztekin et al. used the PSQI score to examine the data separately for men and women to see if sex has a factor in sleep quality decline [22]. PSQI (7.5) scores in the female AA population were found to be higher than PSQI (6) scores in the male patient group (p = 0.035), according to the analyses [22]. In a subgroup analysis, Seo et al. examined the incidence rate of alopecia areata in people who had trouble sleeping [25]. Females had a greater incidence ratio than males (1.42 per 1000 person-years) (1.00 per 1000 person-years) [25]. Male sleeping disorder patients had a higher incidence rate (adjusted HR 1.532 [95% CI 1.112–2.111]) than a control group of male patients with no underlying sleeping difficulties (log-rank p = 0.006) [25]. The female cohort exhibited similar significant outcomes when compared to the control group; adjusted HR 1.695 [95% CI 1.367–2.101]. (log-rank p 0.001) [25].

## 4 Discussion

This is the first systematic review that assesses the bi-directional association between Alopecia Areata (AA) and sleep disorders. With a global prevalence of around 2%, AA is the most prevalent Autoimmune disorder and the second most prevalent hair loss disorder worldwide [4]. There is ample literature regarding the association between AA and psychiatric disorders. However, the link between AA and sleep disorders still remains unclear [15,26]. Therefore, it was imperative that we evaluated this relation between AA and sleep disorders.

In this study, we reviewed a total of 8 observational studies that assessed the bi-directional association between AA and sleep disorders. 6 of these studies evaluated our primary outcome that is the development of sleep disorders in AA patients. The studies by Shakoei et al., Oztekin et al., Kim et al. and Sorour et al. [1,20,22,24] reported significant evidence of sleep disorders and/or reduced sleep quality in patients with AA Another bi-directional cohort by Dai et al. [15] also supported the finding that the risk of developing onset sleep disorders was exacerbated in patients with AA. Conversely, Inui et al. and Kashaninasab et al. [21,23] did not report a significant association between AA and sleep disorders. The secondary outcome of our review pertained to the development of AA in patients with sleep disorders. During the evaluation of our secondary outcome, findings from the 2 observational studies by Seo et al. and Dai et al. [15,25] reported that patients with sleep disorders faced a much greater risk of developing AA as compared to the patients without sleep disorders. Moreover, 2 of the aforementioned studies also assessed the relation of sleep disturbance and quality with severity of Alopecia. Inui et al. and Oztekin et al. [21,22] reported no significant correlation between severity of AA and sleep quality. In addition, findings from 3 studies including Shakoei et al., Oztekin et al. and Seo et al. [20,22,25] revealed that female AA patients were more prone to developing sleep disorders as compared to their male counterparts.

Currently, a significant research gap exists in regards to the incidence and association of Alopecia Areata with sleep disorders. Therefore, there is a dire need for more observational studies that assess and elucidate the aforementioned association. Following the research methodology of Kashaninasab et al. [23], future studies should consider simultaneously evaluating Quality of Life (QOL) and sleep quality in AA patients for a more holistic investigation. Furthermore, increased focus and efforts should be directed towards the early detection of AA for improved management of sleep disorders in affected patients. Similarly, there also needs to be a mechanism for the early detection of sleep disorders to halt or at least limit the development of AA in such patients.

This systematic review has a few limitations. Firstly, most of the studies included in our review had a relatively small study population and there was inadequate data across studies to enable a meta-analysis. The assessment methods used to measure sleep quality varied from study to study and this might have affected the consistency of the results. Moreover, most of the studies included in this review have a cross-sectional design that limits the assessment of causality association between sleep disorders and AA [1]. Similarly, these cross-sectional studies might have underestimated the incidence of AA and sleep disorders since only those who came in for consultation and treatment were included in the studies [15].

## 5 Conclusion

The findings from our results display a significant bi-directional cause-effect relation between AA and sleep disorders. However, more large-scale observational studies on this subject are required to further validate our findings.

## Ethics statement

The present study includes printed and published information; therefore, the formal ethical clearance was not applicable for this study.

## Funding

None.

## Author contribution

STR and MMH: conceived the idea, designed the study, and drafted the manuscript.

STR, ZK, HM, and SHS: conducted the literature search and created the illustrations.

STR and MMH: revised the manuscript critically and refined the illustrations.

MMH, STR, and ZK: revised the final version of the manuscript critically and gave the final approval.

## Registration of research studies

1. Name of the registry: NA
2. Unique Identifying number or registration ID: NA
3. Hyperlink to your specific registration (must be publicly accessible and will be checked): NA

## Guarantor

Mohammad Mehedi Hasan.

Department of Biochemistry and Molecular Biology, Faculty of Life Science, Mawlana Bhashani Science and Technology University, Tangail, 1902, Bangladesh.

Email: mehedi.bmb.mbstu@gmail.com.

## Consent

NA.

## Provenance and peer review

Not commissioned, externally peer reviewed.

## Declaration of competing interest

The authors declare that there is no conflict of interests.
