# Supplementary material for: Two-way association between alopecia areata and sleep disorders: A systematic review of observational studies
Source: Ann Med Surg (Lond). 2022 Nov 5;84:104820. doi: 10.1016/j.amsu.2022.104820 (PMC9793131; doi:10.1016/j.amsu.2022.104820)
Supplement: Multimedia component 3 [file mmc3.docx]

**Table S1.** Detailed Search Strategy.

| **Database** | **String** | **Results** |
| --- | --- | --- |
| PubMed | ("alopecia areata"[MeSH Terms] OR ("alopecia"[All Fields] AND "areata"[All Fields]) OR "alopecia areata"[All Fields] OR ("alopecia"[MeSH Terms] OR "alopecia"[All Fields] OR "alopecias"[All Fields]) OR ("alopecia"[MeSH Terms] OR "alopecia"[All Fields] OR "baldness"[All Fields] OR "balding"[All Fields])) AND ("sleep wake disorders"[MeSH Terms] OR ("sleep"[All Fields] AND "wake"[All Fields] AND "disorders"[All Fields]) OR "sleep wake disorders"[All Fields] OR ("sleep"[All Fields] AND "disorders"[All Fields]) OR "sleep disorders"[All Fields] OR ("sleep wake disorders"[MeSH Terms] OR ("sleep"[All Fields] AND "wake"[All Fields] AND "disorders"[All Fields]) OR "sleep wake disorders"[All Fields] OR ("sleep"[All Fields] AND "disturbance"[All Fields]) OR "sleep disturbance"[All Fields]) OR ("sleep initiation and maintenance disorders"[MeSH Terms] OR ("sleep"[All Fields] AND "initiation"[All Fields] AND "maintenance"[All Fields] AND "disorders"[All Fields]) OR "sleep initiation and maintenance disorders"[All Fields] OR "sleeplessness"[All Fields] OR "sleepless"[All Fields]) OR ("insomnia s"[All Fields] OR "sleep initiation and maintenance disorders"[MeSH Terms] OR ("sleep"[All Fields] AND "initiation"[All Fields] AND "maintenance"[All Fields] AND "disorders"[All Fields]) OR "sleep initiation and maintenance disorders"[All Fields] OR "insomnia"[All Fields] OR "insomnias"[All Fields]) OR ("sleep apnoea"[All Fields] OR "sleep apnea syndromes"[MeSH Terms] OR ("sleep"[All Fields] AND "apnea"[All Fields] AND "syndromes"[All Fields]) OR "sleep apnea syndromes"[All Fields] OR ("sleep"[All Fields] AND "apnea"[All Fields]) OR "sleep apnea"[All Fields]) OR ("obstructive sleep apnoea"[All Fields] OR "sleep apnea, obstructive"[MeSH Terms] OR ("sleep"[All Fields] AND "apnea"[All Fields] AND "obstructive"[All Fields]) OR "obstructive sleep apnea"[All Fields] OR ("obstructive"[All Fields] AND "sleep"[All Fields] AND "apnea"[All Fields])) OR ("sleep quality"[MeSH Terms] OR ("sleep"[All Fields] AND "quality"[All Fields]) OR "sleep quality"[All Fields])) | 87 |
| Cochrane CENTRAL | (((alopecia areata) OR (alopecia*) OR (baldness*)) AND ((sleep disorders) OR (sleep disturbance*) OR (sleeplessness) OR (insomnia) OR (sleep apnea) OR (obstructive sleep apnea) OR (sleep quality))) | 215 |
| Google  Scholar | "alopecia areata" OR "baldness" AND "sleep disturbances" OR "sleep quality" | 1260 |
